# Knowledge of ORS packet or pre-packaged liquids and its determinants for the management of diarrhea among women of reproductive age: multilevel analysis of 32 sub-Saharan African countries demographic and health survey

**DOI:** 10.1186/s41182-022-00477-6

**Published:** 2022-11-01

**Authors:** Fantu Mamo Aragaw, Daniel Bekele Ketema, Maereg Wolde

**Affiliations:** 1 Department of Epidemiology and Biostatistics, Institute of Public Health, College of Medicine and Health Sciences, University of Gondar, Gondar, Ethiopia; 2 Department of Public Health, College of Health Science, Debre Markos University, Debre Markos, Ethiopia; 3 Department of Health Education and Behavioral Sciences, Institute of Public Health, College of Medicine and Health Sciences, University of Gondar, Gondar, Ethiopia

**Keywords:** Knowledge, Multilevel analysis, ORS packet, Sub-Saharan Africa

## Abstract

**Background:**

Infant and child mortality due to diarrhea is a very serious and widespread problem all over the world, particularly in sub-Saharan African countries. Using an oral rehydration solution (ORS) is an easy, inexpensive, and reliable way of treating dehydration and reducing diarrhea-related mortalities. However, there is limited evidence on the magnitude of knowledge of ORS packets or pre-packaged liquids and determinant factors among women in sub-Saharan African countries. Hence, This study sought to assess knowledge of ORS packets or pre-packaged liquids and determinant factors for the management of diarrhea among women of reproductive age in 32 sub-Saharan African countries.

**Method:**

Data for the study were drawn from a recent 32 demographic and health surveys (DHS) conducted in sub-Saharan African countries. A total sample of 234,848 mothers who gave birth in the last 5 years preceding the survey was included. STATA version 16 was used to clean and analyze the data. Multilevel multivariable logistic regression was employed to identify factors associated with knowledge of ORS packets or pre-packaged liquids in sub-Saharan African countries. In the multivariable analysis, an adjusted odd ratio with a 95% confidence level was reported to indicate statistical association with a *P* value < 0.05.

**Results:**

The overall magnitude of knowledge of ORS packets or pre-packaged liquids in sub-Saharan African countries were 80.59% (95% CI: 80.42%, 80.74%). Individual-level factors such as women who were aged 25 -39, (AOR = 1.30; 95%CI; 1.27, 1.34) and aged > 35 (AOR = 1.44; 95%CI; 1.40,1.49),women having primary education (AOR = 1.51; 95%CI; 1.47, 1.56), secondary and above education (AOR = 1.80; 95%CI; 1.74, 1.86), women who were working (AOR = 1.38; 95%CI; 1.35, 1.42), household size of 6–10, & > 10, (AOR = 1.08; 95%CI; 1.05, 1.10) and (AOR = 1.10; 95%CI; 1.06, 1.14), women from middle and rich household (AOR = 1.09 95%CI; 1.06, 1.12) and (AOR = 1.51 95%CI; 1.47, 1.56), media exposure (AOR = 1.20 95%CI; 1.17, 1.23), ANC visit (AOR = 2.11 95%CI; 2.04, 2.17), living in regions of East Africa, West Africa and Southern Africa have 2.45 (AOR = 2.45 95%CI; 2.36, 2.53), 2.21 (AOR = 2.21 95%CI; 2.14, 2.27), 1.95 (AOR = 1.95 95%CI; 1.83, 2.08) were significantly associated with womens knowledge about ORS packet or pre-packaged liquids.

**Conclusion:**

One in five women does not know ORS packets or pre-packaged liquids. Maternal age, women’s education, working status, household wealth index, household size Media exposure, ANC visit, and region were significant predictors of knowledge of ORS packets or pre-packaged liquids. Therefore, it is better to give special emphasis to young age, women who had no formal education and who have no media exposure, poor households, those women who have not currently working as well as those who have a household size of greater than six. Furthermore, it is critical to increase ANC visits to improve knowledge of ORS packets or pre-packaged liquids.

## Introduction

Diarrhea is one of the leading causes of mortality and morbidity among children under the age of five, even though it can be prevented easily [1]. Mortality due to diarrhea among infants and children is a very common and devastating problem throughout the globe, in low- and middle-income countries, and sub-Saharan countries [2–9]. Over three million children under the age of five die of dehydration as a result of diarrhea, an easily preventable and treatable disease [10]. The infection is spread by contaminated food or drinking water, or from person to person as a result of poor sanitation, and it is easily prevented by using clean water, sanitation, and hygiene [2, 3].

The leading cause of death from acute diarrhea is the loss of fluids and mineral elements, which could have been avoided and compensated for by oral rehydration solution (ORS), which is the most beneficial, effective, simple, cost-effective, and reliable treatment for dehydration and lowering diarrhea-related deaths [2, 3, 11–13]. Caregivers can easily manage the treatment and prevention of diarrhea at home using ORS. As a result, it is essential for mothers and primary caregivers to understand strategies for preventing and treating diarrhea [2, 11, 14, 15].

The mother's knowledge of diarrheal disease management remained an important determinant of diarrheal morbidity [16]. However, caregivers stated that their diarrhea management practices were based on advice from others as well as their observations or understanding of the beneficial effects of particular diarrhea treatments [17].

Studies revealed that knowledge of ORS had a significant influence on ORS utilization during diarrhea management [12, 18, 19]. The primary reasons for not using ORS in breastfeeding mothers were a lack of knowledge regarding ORS, as well as information about its composition and preparation [19]. In addition, studies suggest that harmful practices in treating diarrhea such as restricting liquids, breast milk, and/or dietary intake during episodes of diarrhea, and incorrect use of modern medications, are common in some countries, where the burden of diarrheal mortality is high [17].

According to some studies, approximately half of the women were knowledgeable about ORS [14, 18]. Some studies also reported that very few mothers were knowledgeable regarding the use of ORS in the treatment of diarrheal dehydration [4, 5]. Studies also revealed that factors such as maternal education, media exposure, residence, community illiteracy level, and region were significant predictors of knowledge of ORS [12].

Although increasing ORS knowledge among women of reproductive age had a significant contribution to lowering infant and child morbidity and mortality by increasing ORS utilization, there is no study regarding the magnitude and associated factors of knowledge of ORS among women of reproductive age in sub-Saharan Africa as per our knowledge. Thus, this study was aimed at determining the magnitude and associated factors towards knowledge of ORS among women of reproductive age in sub-Saharan Africa.

## Methods

### Study population and data source

The most recently collected standard cross-sectional DHS data set of the SSA countries within 10 years (2010–2020) was used as our data source. The DHS survey is a nationally representative survey that collects data on basic health indicators. Each country's survey includes a variety of datasets such as men, women, children, births, and household datasets. For this study, we used individual record (IR) data.

This study only included women between the ages of 15 and 49 who had given birth within the previous 5 years of the surveys. As a result, the total sample size was 234,848, with respondents from each country ranging from 2064 in Comoros to 21,911 in Nigeria. A detailed description of the survey year, sample size, and sample characteristics is presented in Table 1.

**Table 1:** Sample size determination in the study of magnitude ORS packets or ORS pre-packaged liquids among women of reproductive age, DHS, 2010–2020

Region	Countries	DHS year	Weighted	Percentage (%)
East Africa countries			**82,385**	**35.08**
	Burundi	2016/17	8941	3.81
	Comoros	2012	2064	0.88
	Ethiopia	2016	7590	3.23
	Kenya	2014	14,430	6.14
	Malawi	2015/16	13,515	5.75
	Rwanda	2019/2020	6302	2.68
	Tanzania	2015/16	7078	3.01
	Uganda	2016	10,152	4.32
	Zambia	2018	7325	3.12
	Zimbabwe	2015	4988	2.12
Central Africa countries			**46,576**	**19.83**
	Chad	2014/15	10,887	4.64
	Angola	2015/16	8495	3.62
	Cameroon	2018	6612	2.82
	DR Congo	2013/14	10,998	4.68
	Congo	2011/12	5882	2.50
	Gabon	2012	3702	1.58
West Africa countries			**96,476**	**41.08**
	Benin	2017/18	9031	3.85
	Burkina Faso	2011	10,487	4.47
	Cote d’vore	2011/12	5167	2.20
	Gambia	2019/20	5372	2.29
	Ghana	2014	4141	1.76
	Guinea	2018	5488	2.34
	Liberia	2019/20	4025	1.71
	Mali	2018	6623	2.82
	Niger	2012	8002	3.41
	Nigeria	2018	21,911	9.33
	Senegal	2019	4056	1.73
	Sierra Leone	2019	7326	3.12
	Togo	2013/14	4847	2.06
Southern Africa countries			**9408**	**4.01**
	Lesotho	2014	2574	1.10
	Namibia	2013	3797	1.62
	South Africa	2016	3035	1.29

The surveys are nationally representative of each country and population-based with large sample sizes. All surveys use a multistage cluster sampling method [20].

### Study variables and measurement

Knowledge of ORS packets or pre-packaged liquids was the outcome variable. Women had knowledge regarding ORS packets or pre-packaged liquids if they heard about it or used it to treat diarrhea; otherwise, the women had no knowledge [12].

The study included individual-level factors such as the age of women (15–24, 25–34, > 35 years), educational status of women (no education, primary, and secondary and higher), media exposure (yes or no), working status (working or not working), number of household members (1–5, 6–10, or > 10), and sex of household head (male or female), ANC visit (yes, no). Media exposure was an individual-level factor generated by the aggregation of reading a newspaper, listening to the radio, and watching television and dichotomized as yes "if the mother has exposure to either of the three media sources. The wealth index for the households was also an individual-level factor which was originally classified into five categories by DHS which was done using principal component analysis but for easy understanding, we re-categorized the wealth scores into three categories (poor, medium, and rich).

Place of residence (rural or urban), region (East Africa countries, Central African countries, West African countries, and Southern African countries), and survey year (2010–2014 and 2015–2020) were community-level factors included in the study.

### Data processing and analysis

We combined the data after extracting the variables based on existing literature. The data were weighted using an individual sampling weight for women (v005) divided by 1,000,000 before any statistical analysis to restore the survey’s representativeness. The data were cleaned and statistical analysis was carried out using STATA version 16. Frequencies and percentages were used to describe the background characteristics of the study participants. We used a multilevel logistic regression analysis by assuming that each community has a different intercept and fixed coefficient, the clustered data nature as well as within and between community variations, with a random effect applied at the cluster level. Finally, variables with *p*-values less than 0.2 in crude odds ratio (COR) were selected as candidates for the adjusted model. Finally, the adjusted odd ratio (AOR) with 95% CI was reported, and variables with p-values less than 0.05 were declared to be significant predictors of knowledge of ORS packets or pre-packaged liquids in the multivariable analysis.

Since the DHS data has a hierarchical nature, random effects or measures of variation of the outcome variables were estimated by the median odds ratio (MOR), intraclass correlation coefficient (ICC), and proportional change in variance (PCV). Taking clusters as a random variable, the MOR is defined as the median value of the odds ratio between the area at the highest risk and the area at the lowest risk when randomly picking out two clusters^.^ In contrast, the ICC reveals the variation of knowledge of ORS packets or pre-packaged liquids between clusters. Moreover, the PCV reveals the variation in the prevalence of knowledge of ORS packets or pre-packaged liquids among women of reproductive age.

Four models have fitted: the null model (models without independent variables), model I (models with individual-level variables), model II (models including community-level variables), and model III (models with both individual and community-level variables). Deviance was used to assess model fitness, since these models were nested. Model III, which includes both individual and community-level variables, was selected as the best-fitted model since it had a low deviance value (211,720) (Table 3). The results of the random effect were estimated using different methods such as intra-class correlation (ICC), median odds ratio (MOR), and proportional change in variance (PCV).

### Ethics consideration

The data set was obtained from the DHS website after formal requests and permission from the major DHS. All methods were performed following the demographic and health surveys (DHS) program’s relevant guidelines and regulations. The data set was not allowed to be shared with other organizations and has remained confidential.

## Results

### Sociodemographic characteristics of the study population

Total weighted sample of 234,848 households were included in this study, Nearly, half of the study participants were found in the age group 25–34 years (108,276 (46.11%). Most of the study participants, 154,546 (65.81%), lived in rural areas, and around 86,970 (37.03%) had no formal education (Table 2).

**Table 2:** Knowledge of ORS packets or pre-packaged liquids for the management of diarrhea across variables among women of reproductive age in sub-Saharan Africa, DHS 2010–2020

Variables	Categories	Knowledge of ORS packets or pre-packaged liquids		Total weighted frequency (%)
		Yes (%) n = 189,261 (80.59)	No (%) n = 45,586 (19.41)	
Age of women	15–24	53,648 (77.27)	15,777 (22.73)	69,425 (29.56)
	25–34	88,554 (81.78)	19,722 (18.22)	108,276 (46.11)
	> 35	47,059(82.35)	10,086(17.65)	57,145(24.33
Women education status	No education	64,473 (74.13)	22,496(25.87)	86,970 (37.03)
	Primary	63,499 (83.16)	12,856 (16.84)	76,355 (32.51)
	Secondary and higher	61,288 (85.69)	10,233(14.31)	71,522 (30.45)
Wealth index	Poor	75,500 (76.08)	23,744 (23.92)	99,244 (42.26)
	Middle	37,447 (79.69)	9,544 (20.31)	46,992 (20.01)
	Rich	76,314 (86.12)	12,297 (13.88)	88,611 (37.73)
Occupation of women	Not working	47,204 (75.16)	15,604 (24.84)	62,808 (26.74)
	Working	142,056 (82.57)	29,982 (17.43)	172,039 (73.26)
Household size	1–5	83,940 (80.68)	20,096 (19.32)	104,037 (44.30)
	6–10	81,747 (80.69)	19,563( 19.31)	101,310 (43.14)
	> 10	23,573(79.91)	5,927 (20.09)	29,500 (12.56)
Sex of household head	Male	148,912 (80.48)	36,127( 19.52)	185,040 (78.79)
	Female	40,348 (81.01)	9,459 (18.99)	49,807 (21.21)
Media exposure	No	60,033 (74.20)	20,871 (25.80)	80,905 (34.50)
	Yes	128,984 (83.98)	24,613 (16.02)	153,598 (65.50)
ANC visit	No	17,761 (62.61)	10,607 (37.39)	28,368 (12.08)
	Yes	171,499 (83.06)	34,978 (16.94)	206,478 (87.92)
Community level variables				
Residence	Urban	67,305 (83.82)	12,995 (16.18)	80,301 (34.19)
	Rural	121,955 (78.91)	32,590 (21.09)	154,546 (65.81)
Region	East Africa	70,831 (85.98)	11,553 (14.02)	82,385 (35.08)
	West Africa	78,630 (81.50)	17,845 (18.50)	96,476 (41.08)
	Central Africa	31,955 (68.61)	14,621 (31.39)	46,576 (19.83)
	Southern Africa	7843 (83.36)	1565 (16.64)	9408 (4.01)
Survey year	2010–2014	70,717 (77.68)	20,320 (22.32)	91,038 (38.76)
	2015–2020	118,544 (82.43)	25,265 (17.57)	143,809 (61.24)

### Prevalence of knowledge of ORS packets or pre-packaged liquids

The overall magnitude of knowledge of ORS packets or pre-packaged liquids in sub-Saharan African countries was 80.59% (95% CI: 80.42%, 80.74%) (Table 2).

### Random effect and model comparison

As indicated in Table 3, the ICC in the null model was 0.11, which means that about 11% of the variations in knowledge of ORS packets or pre-packaged liquids among study subjects were attributed to the difference at the cluster level, but the rest 89% were attributed to individual women’s factors.

**Table 3:** Model fitness and random effect measures

Measure of variation	Null model	Model I	Model II	Model III
VA	0.41	0.33	0.30	0.29
ICC	0.11	0.09	0.084	0.082
MOR	1.65	1.48	1.41	1.38
PCV (%)	–	24%	26.8%	29.2%
Model fitness				
Deviance	227,339	215,733	220,581	211,720

*ICC* inter cluster corrolation cofficent, *MOR* median odds ratio, *PCV* proportional change in variance

Furthermore, the PCV value, 0.292, in the final model indicates that about 29.2% of the variations in knowledge of ORS packets or pre-packaged liquids among study subjects were attributed to both individual and community-level factors. Regarding model comparison and fitness, deviance was used. The model with the lowest deviance was the best-fitted model, which was model three (211,720) (Table 3).

### Multi-level analysis of factors associated with knowledge of ORS packet or pre-packaged liquids among women of reproductive age

All variables that had a *P*-value of 0.20 in the bivariable analysis were eligible for multivariable analysis. Based on the final model result, the age of the women, women’s educational status, working status of women, household head, media exposure, household size, ANC visit, household wealth index, and region have a significant association with knowledge of ORS packet or pre-packaged liquids.

Women who were aged 25–39 had 1.30 (AOR = 1.30; 95%CI; 1.27, 1.34), and those aged > 35 had 1.44 (AOR = 1.44; 95%CI; 1.40, 1.49) times higher odds of knowledge of ORS packet or pre-packaged liquids as compared to women aged 15–24. Women who have primary education were 1.51 (AOR = 1.51; 95%CI; 1.47, 1.56), and secondary and higher education were 1.80 (AOR = 1.80; 95%CI; 1.74, 1.86) times more likely to have knowledge of ORS packets or pre-packaged liquids as compared to women who have no education.

Women who were currently working had 1.38 (AOR = 1.38; 95%CI; 1.35, 1.42) times higher odds of knowledge of ORS packets or pre-packaged liquids as compared to women who were not currently working. Women with a household size of 6–10 & > 10 have 1.08 (AOR = 1.08; 95%CI; 1.05, 1.10) and 1.10 (AOR = 1.10; 95%CI; 1.06, 1.14) times higher odds of knowledge of ORS packet or pre-packaged liquids as compared to women who have a household size of 1–5, respectively.

Women with middle and rich household wealth index were 1.09 (AOR = 1.09 95%CI; 1.06, 1.12) and 1.51 (AOR = 1.51 95%CI; 1.47, 1.56) times higher odds of knowledge of ORS packet or pre-packaged liquids as compared to women with poor household wealth index respectively.

Those women who were media exposed had 1.20 (AOR = 1.20, 95%CI; 1.17, 1.23) times higher odds of knowledge of ORS packets or pre-packaged liquids as compared to women without media exposure. Those women who had an ANC visit had 2.11 (AOR = 2.11, 95%CI; 2.04, 2.17) times higher odds of knowledge of ORS packet or pre-packaged liquids as compared to women who had no ANC visit. Those women who live in regions of East Africa, West Africa, and Southern Africa have 2.45 (AOR = 2.45 95%CI; 2.36, 2.53), 2.21 (AOR = 2.21 95%CI; 2.14, 2.27), and 1.95 (AOR = 1.95 95%CI; 1.83, 2.08) times higher odds of knowledge of ORS packets or pre-packaged liquids as compared to women who live in regions of Central Africa, respectively (Table 4).

**Table 4:** Multilevel multivariable analysis of factors associated with knowledge of ORS packet or pre-packaged liquids for the management of diarrhea among women of reproductive age in sub-Saharan Africa, DHS 2010–2020

Variables	Categories	Null model	Model I	Model II	Model III
			AOR [95% CI]	AOR [95% CI]	AOR [95% CI]
Age of women	15–24		1.00		1.00
	25–34		1.32 [1.29, 1.35]*		1.30 [1.27, 1.34]*
	> 35		1.47 [1.43, 1.52]*		1.44 [1.40, 1.49]*
Women education status	No education		1.00		1.00
	Primary		1.43 [1.39, 1.47]*		1.51 [1.47, 1.56]*
	Secondary and Higher		1.52 [1.48, 1.57]*		1.80 [1.74, 1.86]*
Occupation of women	Not working		1.00		1.00
	Working		1.41 [1.38, 1.44]**		1.38 [1.35, 1.42]**
Household size	1–5		1.00		1.00
	6–10		1.03 [1.01, 1.06]*		1.08 [1.05, 1.10]*
	> 10		1.05 [1.02, 1.09]*		1.10 [1.06, 1.14]*
ANC visit	No		1.00		1.00
	Yes		2.34 [2.28, 2.41]*		2.11 [2.04, 2.17]*
Sex of household head	Male		1.00		1.00
	Female		0.97 [0.94, 1.00]		0.98 [0.95, 1.01]
Wealth index	Poor		1.00		1.00
	Middle		1.07 [1.04, 1.10]*		1.09 [1.06, 1.12]*
	Rich		1.49 [1.45, 1.53]*		1.51 [1.47, 1.56]*
Media exposure	No		1.00		1.00
	Yes		1.32 [1.29, 1.35]***		1.20 [1.17, 1.23]***
Community-level variables					
Residence	Rural			1.00	1.00
	Urban			1.63 [1.59, 1.67]*	0.98 [0.95, 1.01]
Region	Central Africa			1.00	1.00
	East Africa			3.14 [3.04, 3.24]*	2.45 [2.36, 2.53]*
	West Africa			2.19 [2.13, 2.25]*	2.21 [2.14, 2.27]*
	Southern Africa			2.38 [2.24, 2.52]*	1.95 [1.83, 2.08]*
Survey year	2010–2014			1.00 [0.98, 1.02]	0.98 [0.95, 1.00]
	2015–2020			1.00	1.00

*P value < 0.05, **P value < 0.01, ***P value < 0.001

*AOR* adjusted odds ratio, *CI* confidence interval

## Discussion

A woman's age has a significant association with knowledge of ORS packets or pre-packaged liquids. Those women aged 25–34 and > 35 had higher odds of knowledge of ORS packets or pre-packaged liquids as compared to women aged 15–24. This finding is supported by a study done in Iran [21], India [22], and Ethiopia [23]. The possible reason might be that older women may have more children, which may lead them to have previous experience, so they may have had better knowledge about diarrhea management [21].

Women having primary education and those with secondary and above education had higher odds of knowledge of ORS packets or pre-packaged liquids as compared to women who have no education. This finding is similar to a study done in Ethiopia [12], India [24, 25], and Iran [2]. The possible explanation might be that women with higher education are more likely to be aware of effective diarrhea management mechanisms such as the ORS packet or pre-packaged liquids.

Media exposure was associated with knowledge of ORS packets or pre-packaged liquids. Women who had been exposed to the media had higher odds of knowledge of ORS packets or pre-packaged liquids as compared to their counterparts. This is in line with a study in India [26], and Ethiopia [12]. The possible reason might be that mothers who have had media exposure have been exposed to diarrhea management approaches for children and will have knowledge of ORS packets or pre-packaged liquids.

Women currently working had higher odds of knowledge of ORS packets or pre-packaged liquids as compared to those who were not currently working. This finding is supported by a study done in Iran [21], and Nepal [4]. One possible explanation is that women who are currently working might have been educated, which is directly related to their level of awareness [27].

Those women having an ANC visit had higher odds of knowledge of ORS packets or pre-packaged liquids as compared to those women who had not had an ANC visit. The finding is similar to a study done in Ethiopia [28]. The possible explanation is that women who have ANC visits have access to a wide range of health services, including information on health promotion and prevention. Hence, information obtained from health workers may help increase women’s knowledge and foster a positive attitude toward the cause, prevention, and management of diarrhea, including the use of ORS packets or pre-packaged liquids [16].

Those women having middle and rich household wealth indexes had higher odds of knowledge of ORS packets or pre-packaged liquids than those women having poor household wealth indexes. The possible justification is that women in middle and upper-income households have access to information and may have good knowledge and practice with the ORS packet. Women having household sizes of 6–10 & > 10 had higher odds of knowledge of ORS packets or pre-packaged liquids than those women having a household size of 1–5. The finding is similar to a study done in Nepal [4], and Iran [21]. The possible justification might be care-seeking pattern was related to the number of children and mothers with previous experience related to their knowledge about the management of diarrhea [21]. In addition, those women living in regions of east, west, and southern Africa had higher odds of knowledge of ORS packets or pre-packaged liquids as compared to those women living in regions of central Africa. The possible reason might be the low healthcare service utilization among women living in central Africa as compared to other regions which makes them have low knowledge of ORS packets or pre-packaged liquids [29]. The other possible reason for the observed difference might be due to the variation in socio-cultural variations, including differences in women's educational attainment in each region.

This study had strengths as well as limitations. Since it is based on nationally representative data, it is appropriate for giving direction to policymakers and program planners to plan intervention strategies. However, it is difficult to investigate causality among dependent and independent variables due to the cross-sectional nature of the data.

## Conclusion

One in five women does not know ORS packets or pre-packaged liquids. Maternal age, women’s education, working status, household wealth index, household size, media exposure, ANC visit, and region were significant predictors of knowledge of ORS packets or pre-packaged liquids. Therefore, it is better to give special emphasis to young age, women who had no formal education and who have no media exposure, poor households, those women who have not currently working as well as those who have a household size of 6–10 & > 10. Furthermore, it is critical to increase ANC visits to improve knowledge of ORS packets or pre-packaged liquids.

## Data Availability

Data are available online and can be accessed from www.measuredhs.com.

## Abbreviations

ANC: Antenatal care
AOR: Adjusted odds ratio
CI: Confidence interval
COR: Crude odds ratio
CSA: Central statistical agency
DHS: Demographic and health survey
ICC: Intraclass correlation coefficient
IR: Individual record
MOR: Median odds ratio
ORS: Oral rehydration solution
PCV: Proportion of change in variance
VA: Variance of the area level
VIF: Variance inflation factors
